# Multiparametric CMR assessment of RV apical versus septal Pacing Study (MAPS) - preliminary acute hemodynamic findings

**DOI:** 10.1186/1532-429X-15-S1-O85

**Published:** 2013-01-30

**Authors:** Mark Ainslie, Christopher A Miller, Benjamin Brown, Neil Davidson, David J Fox, Matthias Schmitt

**Affiliations:** 1 Cardiology, University Hospital of South Manchester, Manchester, UK

## Background

Controversy remains with regards to the optimal site for right ventricular (RV) pacing, with mounting evidence that RV apical pacing leads to accelerated deterioration in left ventricular systolic function both on the background of normal as well as already impaired ventricles. RV septal pacing is conceptually associated with a more physiological activation pattern and as such should confer advantages over apical pacing. However, echocardiographic studies regarding its benefit have been conflicting. The recent introduction of MR-conditional pacing systems, without iso-centre restriction, allows investigation of this fundamental clinical question with the more accurate and reproducible modality of CMR. MAPS is a prospective double blinded cross-over study using multiparametric CMR to determine acute and intermediate effects of RV apical versus septal pacing in patients post atrio-ventricular (AV) node ablation for AF. Here we present preliminary findings of the first acute studies.

## Methods

Eleven patients undergoing AV node ablation and pacemaker implantation as a treatment for atrial fibrillation were prospectively recruited. Each patient received a MR-conditional pacing system (St Jude Accent) with 2 ventricular leads (Tendril MR); one placed at the RV apex and one placed at the mid-septum/RVOT transition. The AV node ablation ensured 100% pacing. CMR (1.5T) was performed 13+/- 1 week after implantation and included aortic and pulmonic phase-contrast velocity mapping during both apical and septal pacing modes. The maximum delay in time to peak strain of opposing wall segments was calculated from 4chamber SSFP cine images using an endocardial feature tracking software package (Diogenes®, Tomtec) and used as a marker for dys-synchrony.

## Results

6 patients were female, average age of the cohort was 68±7. 3. There was trend towards higher mean aortic forward flow volume during septal pacing (72.7±18.3 ml) compared to apical pacing (67.2±13 ml; p=0.083). Likewise, there was a trend towards higher mean pulmonic forward flow volume during septal pacing (112.4±41.6mL) compared to apical pacing (102.7 ±27.1 ml; p=0.14). These hemodynamic improvements occurred on the background of a significant reduction in maximum time to peak strain of opposing wall segments for septal pacing (162 ms ±119 ms) compared to apical (246 ms ±110 ms) pacing (p=0.017).

## Conclusions

These preliminary data suggest that RV septal pacing, by means of a more physiological electromechanical activation pattern, leads to acute hemodynamic benefits. The on-going MAPS trial promises to provide answers with respect to acute and intermediate term benefits in a sufficiently powered and appropriate study design.

## Funding

The MAPS study is supported by a restricted Grant from St Jude. Dr Ainslie is supported by St Jude Medical. Dr Miller is supported by the National Institute of Health Research. Dr Schmitt is supported by the Greater Manchester Comprehensive Local Research Network funding.

